# Endoscopic ultrasound-guided internalization of a pancreaticocutaneous fistula utilizing a balloon-target technique: A case report

**DOI:** 10.1097/MD.0000000000013564

**Published:** 2018-12-14

**Authors:** Michitaka Imai, Yoshifumi Takahashi, Toshihiro Sato, Masaki Maruyama, Osamu Isokawa

**Affiliations:** Department of Gastroenterology, Kashiwazaki General Hospital and Medical Center, Kashiwazaki, Niigata. Japan.

**Keywords:** balloon-target technique, endoscopic ultrasound, pancreaticocutaneous fistula, walled-off pancreatic necrosis

## Abstract

**Rationale:**

Endoscopic ultrasound (EUS)-guided treatment has been recently described for internalizing refractory pancreaticocutaneous fistulas (PCFs). However, the existing techniques are limited because of the difficulty in accessing nondilated pancreatic ducts or fistulas. In an attempt to overcome this limitation, we present a case where a EUS-guided intervention utilizing a balloon-target technique was employed to internalize a PCF into the stomach.

**Patient concerns:**

A 78-year-old woman underwent percutaneous drainage and 4 percutaneous endoscopic necrosectomies for walled-off pancreatic necrosis (WOPN) after severe acute pancreatitis due to choledocholithiasis. Although the WOPN was resolved, refractory PCFs remained.

**Diagnosis:**

Pancreaticocutaneous fistulas.

**Interventions:**

An echoendoscope was introduced into the stomach, but the narrow PCF lumen made visualization of the fistula by EUS difficult. Subsequently, a balloon catheter was percutaneously inserted into the fistula, and then the inflated balloon was visualized by EUS from the stomach. The balloon was punctured with a 19-gauge fine needle through the posterior wall of the upper body of the stomach (balloon-target technique). A guidewire was then passed through the fistula to the outside of the body through the EUS scope. After dilating the gastro-fistula space with an 8-mm balloon dilation catheter, a 7-French double pigtail catheter was placed from the stomach into the PCF.

**Outcomes:**

The percutaneous drainage tube was removed after one week, and the patient was discharged 6 months after admission. No adverse outcomes have been observed in the 2 years since the procedure.

**Lessons:**

PCFs can be successfully managed using EUS-guided internalization with a balloon-target technique.

## 1 Introduction

Acute necrotizing pancreatitis has been reported in approximately 20% of pancreatitis episodes and has a mortality rate of 10% to 30%. Walled-off pancreatic necrosis (WOPN) occurs in 1%–9% of acute pancreatitis cases, usually 4 to 6 weeks after the initial episode. Without procedural intervention,^[1]^ the condition is almost always fatal. Severe necrotizing pancreatitis and WOPN can lead to complete duct disruption and subsequent main pancreatic duct disconnection.^[2,3]^ When percutaneous drainage and necrosectomy are performed for WOPN management, pancreaticocutaneous fistulas (PCFs) can develop from the upstream pancreatic duct through the drain site after the drainage has been completed. The standard endoscopic method for evaluating and treating PCFs is endoscopic retrograde cholangiopancreatography (ERCP).^[4,5]^, but this may fail due to impassable ductal strictures or the inability to effectively bridge a pancreatic duct disruption with a stent. Recently, endoscopic ultrasound (EUS)-guided treatments have been described for internalizing refractory PCFs.^[6,7]^ However, the existing techniques are limited due to difficulty in accessing non-dilated pancreatic ducts or fistulas. Here, we present a case where this limitation was overcome by employing an EUS-guided intervention with a balloon-target technique, providing an alternative drainage pathway that allowed the cutaneous fistula to heal.

## 2 Case presentation

**Figure 1:**
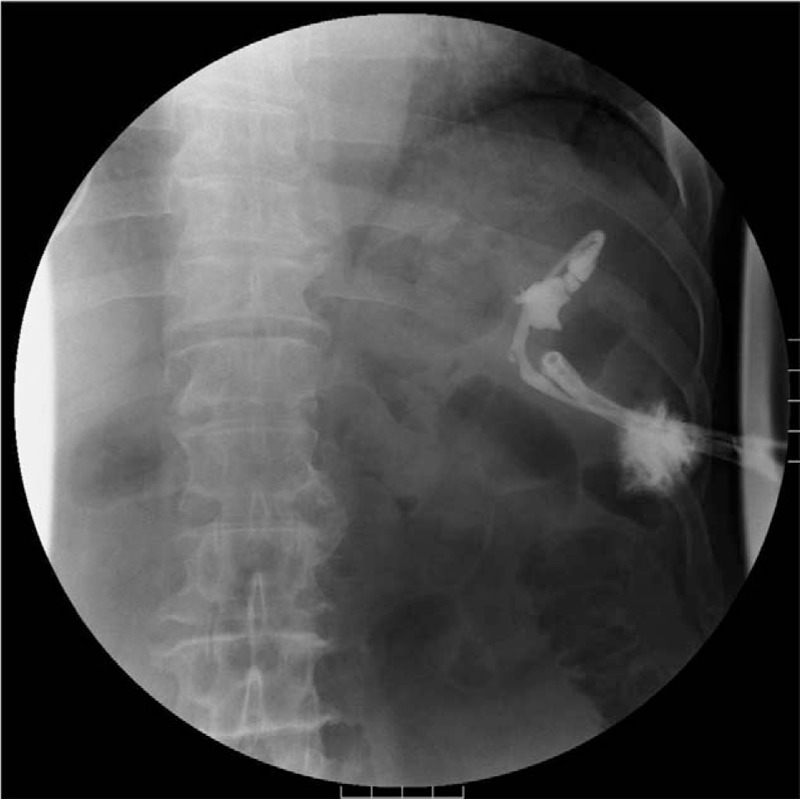
Fluoroscopic image. Fluid collection in the pancreaticocutaneous fistula was visualized by injecting a contrast dye via the percutaneous catheter.

**Figure 2:**
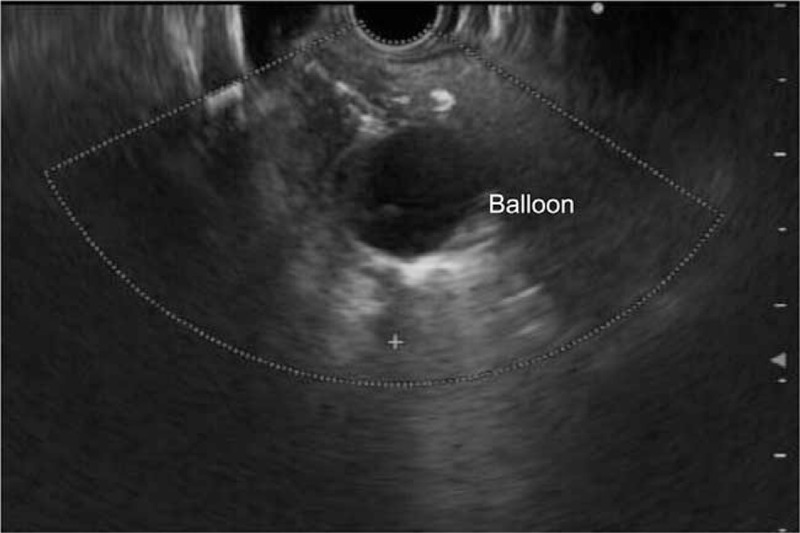
Endosonographic image. The inflated balloon inside the narrow fistula tract.

**Figure 3:**
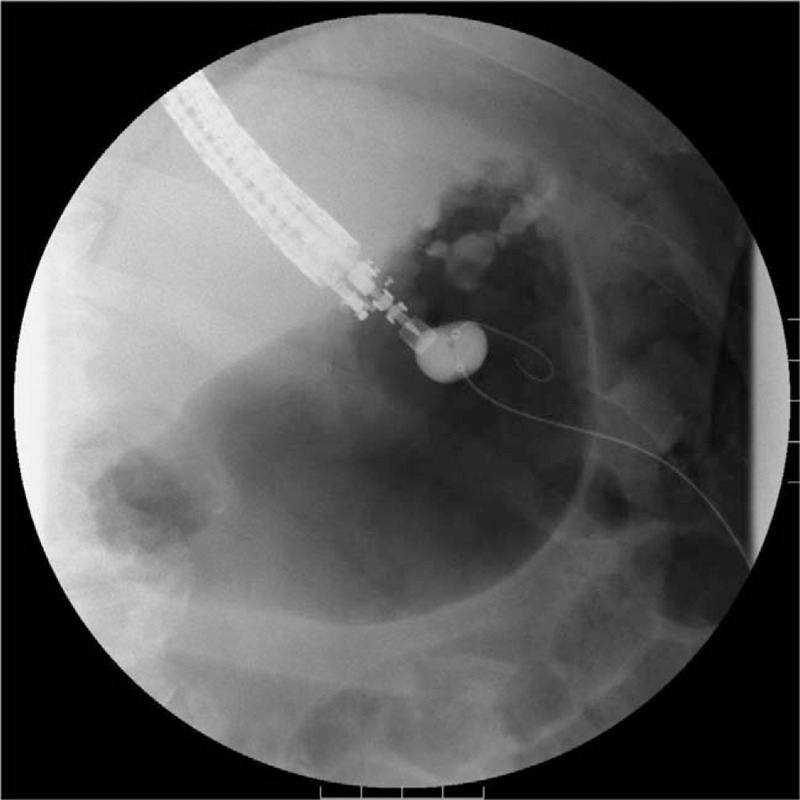
Fluoroscopic image. The balloon-target puncture under endosonographic guidance.

**Figure 4:**
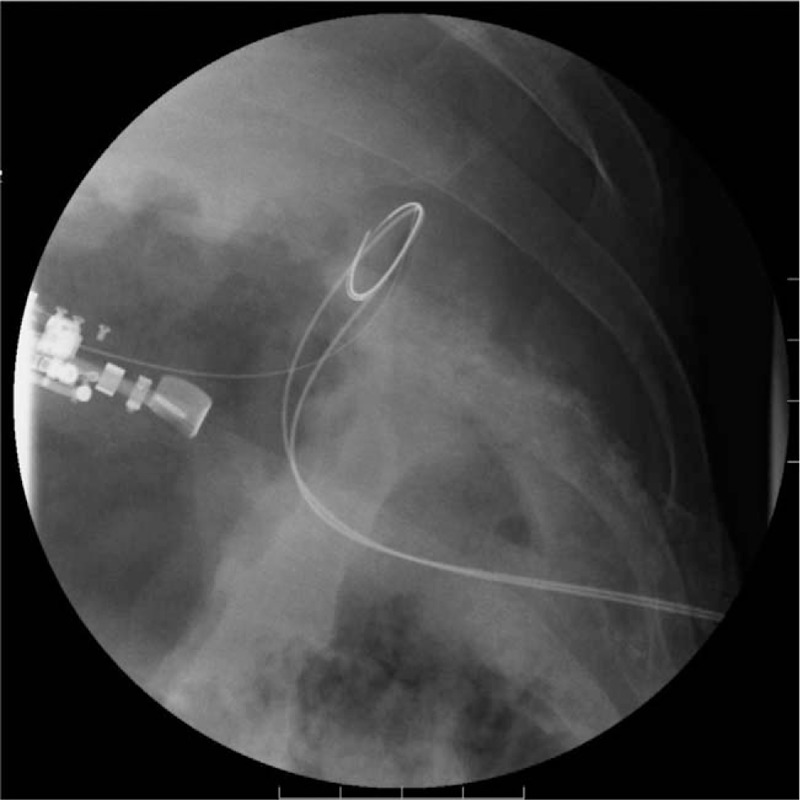
Fluoroscopic image. Guidewire insertion through the endoscopic ultrasound scope into the fistula cavity.

**Figure 5:**
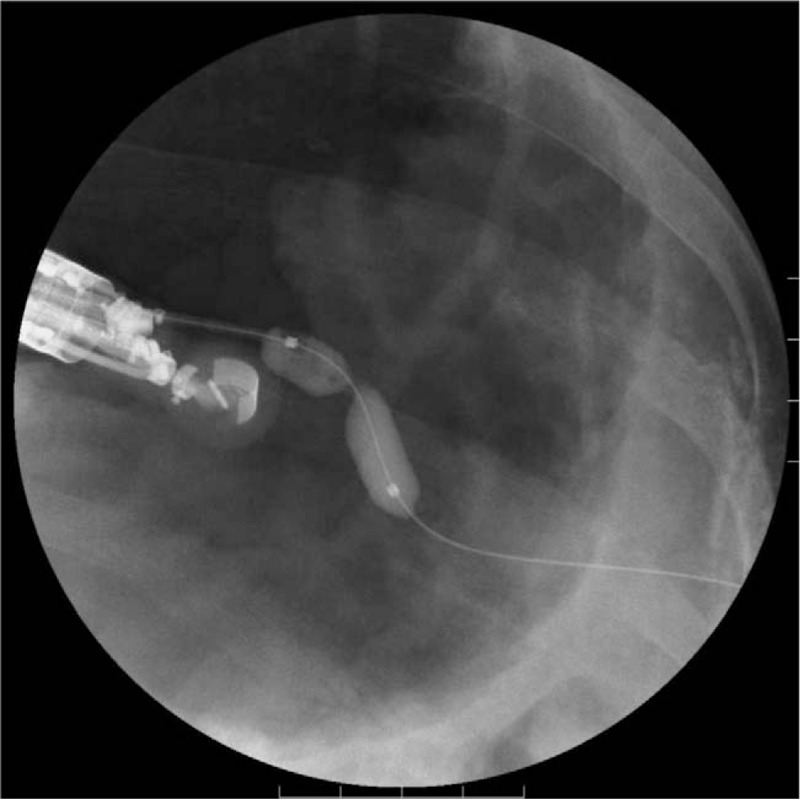
Fluoroscopic image. Dilation of the gastro-fistula space with an 8-mm balloon.

**Figure 6:**
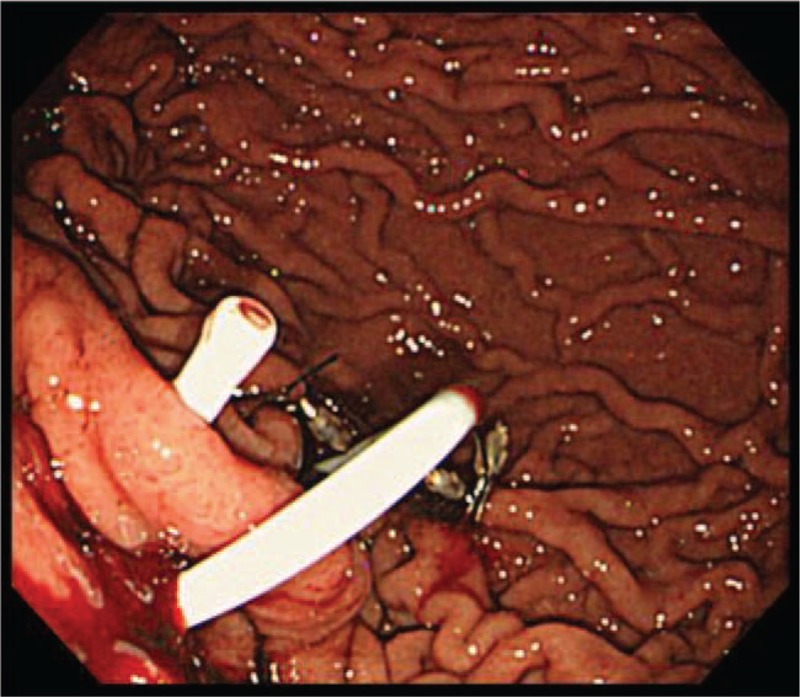
Endoscopic image. Stenting with 7-French double pigtail catheter from the stomach into the pancreaticocutaneous fistula.

**Figure 7:**
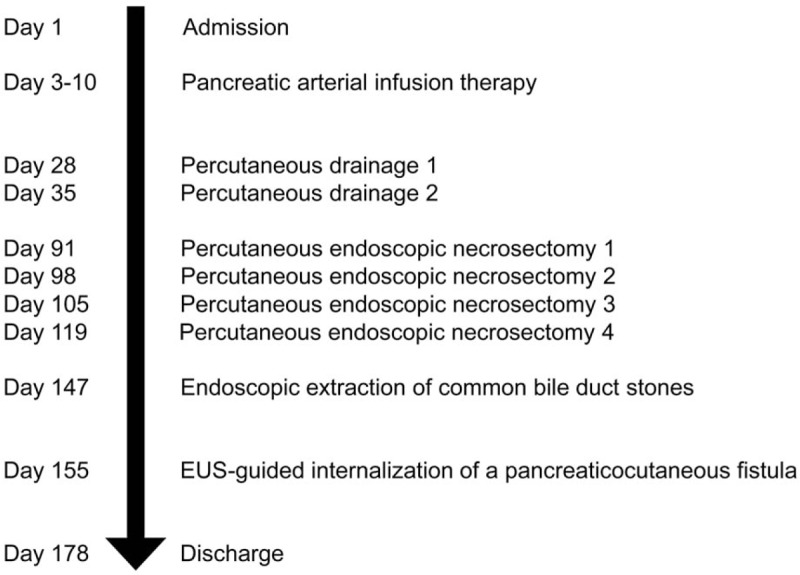
Timeline depicting the clinical course of the patient.

A 78-year-old woman arrived at our hospital by ambulance with severe epigastric pain and vomiting at rest. Her medical history was unremarkable, and she was not receiving any oral medication. Elevated serum amylase levels of 2991 IU/L (reference range 44–132 IU/L) and serum trypsin levels of 8465 ng/mL (reference range 100–550 ng/mL), suggested acute pancreatitis. An increased white blood cell count of 23,900/μL (reference range 3300–8600/μL), indicated severe inflammation. Contrast-enhanced CT revealed pancreatomegaly, effusion extending from the peripancreatic space to the pelvic cavity, and calcified stones in the lower portion of the common bile duct. The patient was diagnosed with acute pancreatitis due to gallstones and was admitted for treatment. An APACHE II score of 12 led to the diagnosis of severe acute pancreatitis.^[8]^ Treatment with continuous intravenous infusion of nafamostat mesilate (240 mg/day), intravenous infusion of meropenem (2.0 g/day), and intravenous infusion of approximately 5000 mL/day was initiated. Two days later, a re-examination CT scan showed a lack of arterial enhancement from the pancreatic body to tail, and pancreatic arterial infusion therapy was administered for 1 week. Four weeks after admission, the patient developed pyrexia of approximately 40°C, and WOPN was suspected on CT scan. Accordingly, percutaneous drainage was performed from the left intercostal space, and a 12-French pigtail drainage catheter (Hanaco Medical, Saitama, Japan) was placed. However, drainage of the necrotic material was insufficient, and a percutaneous endoscopic necrosectomy was performed for WOPN 3 months after admission. The WOPN was resolved after 4 necrosectomies, and endoscopic extraction of the common bile duct stones was performed. Unfortunately, the PCF remained (Fig. 1), and approximately 250 mL of pancreatic fluid was collected daily via percutaneous drainage. Conservative treatments such as total parenteral nutrition and octreotide were ineffective. ERCP revealed complete main pancreatic duct obstruction, which could not be crossed with a guidewire. It was decided to use EUS to internalize the PCF into the stomach. The echoendoscope (GF-UCT260; Olympus Medical Systems, Tokyo, Japan) was carefully introduced into the stomach, but because of the narrow PCF lumen, fistula visualization was difficult. This was overcome by inserting a balloon catheter (Multi-3 V Plus; Olympus Medical Systems) percutaneously into the fistula, and the inflated balloon was visualized by EUS from the stomach (Fig. 2). Then, the balloon was punctured with a 19-gauge fine needle (SonoTip Pro Control; Medi-Globe GmbH, Rosenheim, Germany) through the posterior wall of the upper body of the stomach (balloon-target technique) (Fig. 3). A 0.025-inch guidewire (VisiGlide 2; Olympus Medical Systems) was passed through the fistula to the outside of the body through the EUS scope (Fig. 4). After the gastro-fistula space was dilated with an 8-mm Hurricane RX Biliary Balloon Dilatation Catheter (Boston Scientific, Tokyo, Japan) (Fig. 5), a 7-French double pigtail catheter (Zimmon Biliary Stent; Cook Medical, Tokyo, Japan) was placed from the stomach into the PCF (Fig. 6). The percutaneous drainage tube was removed after one week, and the patient was discharged 6 months after admission. Since postoperative 2 years, no adverse outcomes such as the relapse of acute pancreatitis or appearance of new pancreatic fluid collection have been reported. A timeline depicting the clinical course of the patient is presented in Figure 7.

## 3 Discussion

Severe necrotizing pancreatitis and WOPN can result in complete duct disruption, causing disconnected pancreatic duct syndrome with a viable upstream pancreas segment draining out of a low-pressure fistula created surgically or by a percutaneous catheter.^[2]^ External pancreatic fistulas, known as PCFs, generally result from the placement of a percutaneous drain to collect pancreatic fluid, as occurs in WOPN treatment.^[3]^ The fistula is likely to persist if there is complete transection of the pancreatic duct, a downstream ductal stricture, or if it is a high-output fistula (>500 mL/day). Nonsurgical therapies for pancreatic fistulas include bowel rest, total parenteral nutrition, jejunal feeding, and somatostatin analogs such as octreotide. However, there is limited evidence to support these interventions.^[9,10]^

ERCP is the primary endoscopic modality for PCF treatment, and the placement of a pancreatic duct stent that bridges the ductal disruption often successfully resolves the fistula.^[4,5]^ However, ERCP may fail in patients with disconnected pancreatic duct syndrome where a segment of the pancreatic duct in the tail is completely disconnected from the downstream duct in the pancreatic head. The management options for these patients are long-term percutaneous drain placement or surgery, both associated with substantial morbidity.^[11]^ The access to pancreatic ducts or fistulas via EUS guidance enables the treatment of PCFs that are not amenable to ERCP.^[6,7]^ This EUS-guided rendezvous technique is carried out by the puncture of an EUS needle into the pancreatic duct followed by the passage of a guidewire via the needle and across the ductal obstruction to the gut lumen, facilitating pancreatic stent placement via ERCP.^[12]^ Similarly, EUS-guided transmural stent placement involves positioning a pancreatic guidewire via an EUS needle.^[13]^ The area from the transgastric or transduodenal tract to the pancreatic ducts or fistulas is then dilated, and a stent is placed via the tract. Irani et al^[14]^ pioneered a combined EUS and interventional radiologic technique for the placement of internal stents in PCF patients, in which the fistula tract rather than the pancreatic duct is drained, and much of the manipulation is performed percutaneously with endoscopic or EUS guidance. If the pancreatic duct is dilated or the pancreatic fistula is large, an EUS-guided puncture from stomach or duodenum is easier. However, the pancreatic duct is frequently decompressed by the fistula and usually narrow, preventing access for fluid collection. This paper presents a transgastric balloon-target technique for accessing narrow PCFs using EUS, allowing for placement of a drain from the PCF to the stomach. If the PCF is narrow, a balloon can be placed into the fistula percutaneously, providing a target for EUS-guided puncture and fistula drainage. The balloon placed in the fistula can be clearly visualized by EUS, allowing an easier and safer EUS-guided puncture. One technical challenge generally encountered is difficulty in performing EUS-guided puncture when the blood vessel or intestinal tract is positioned between a fistula and the stomach.

## 4 Conclusion

In conclusion, PCF can be successfully managed using EUS-guided internalization with a balloon-target technique. Creating an alternative drainage pathway from the pancreatic fistula into the stomach diverts pancreatic fluid away from the PCF, allowing healing and obviating the need for long-term percutaneous drain placement or surgery.

## Acknowledgments

We thank the staff of the endoscopy at the Kashiwazaki General Hospital and Medical Center for cooperating in the treatment.

## Author contributions

**Conceptualization:** Michitaka Imai.

**Formal analysis:** Michitaka Imai.

**Investigation:** Michitaka Imai, Yoshifumi Takahashi.

**Methodology:** Michitaka Imai, Toshihiro Sato.

**Supervision:** Michitaka Imai.

**Validation:** Michitaka Imai, Masaki Maruyama.

**Writing – original draft:** Michitaka Imai.

**Writing – review & editing:** Osamu Isokawa.
